# Predicting the potential distribution of 12 threatened medicinal plants on the Qinghai‐Tibet Plateau, with a maximum entropy model

**DOI:** 10.1002/ece3.11042

**Published:** 2024-02-15

**Authors:** Lucun Yang, Xiaofeng Zhu, Wenzhu Song, Xingping Shi, Xiaotao Huang

**Affiliations:** ^1^ Qinghai Province Key Laboratory of Qinghai‐Tibet Plateau Biological Resources, Northwest Institute of Plateau Biology Chinese Academy of Sciences Xining China; ^2^ Gande County Animal Disease Prevention and Control Center Gande Qinghai China; ^3^ Qing Hai West Copper Co., Ltd Maqin Qinghai China; ^4^ School of Geographical Sciences and Tourism Zhaotong University Zhaotong Yunnan China

**Keywords:** barycenter migration, climate change, Qinghai‐Tibet Plateau, suitable habitat, threatened medicinal plants

## Abstract

Climate change is a vital driver of biodiversity patterns and species distributions, understanding how organisms respond to climate change will shed light on the conservation of endangered species. In this study, the MaxEnt model was used to predict the potential suitable area of 12 threatened medicinal plants in the QTP (Qinghai‐Tibet Plateau) under the current and future (2050s, 2070s) three climate scenarios (RCP2.6, RCP4.5, RCP8.5). The results showed that the climatically suitable habitats for the threatened medicinal plants were primarily found in the eastern, southeast, southern, and some parts of the central regions on the QTP. Moreover, 25% of the threatened medicinal plants would have reduced suitable habitat areas within the next 30–50 years in the different future global warming scenarios. Among these medicinal plants, RT (*Rheum tanguticum*) would miss the most habitat (98.97%), while the RAN (*Rhododendron anthopogonoides*) would miss the least habitat (10.15%). Nevertheless, 33.3% of the threatened medicinal plants showed an increase in their future habitat area because of their physiological characteristics which are more adaptable to a wide range of climates. The climatic suitable habitat for 50% of the threatened medicinal plants would migrate to higher altitudes or higher latitudes regions. This study provides a data foundation for the conservation of biodiversity and wild medicinal plants on the QTP.

## Graphical abstract

In this study, the MaxEnt model was used to predict the potential suitable area of 12 threatened medicinal plants in the QTP (Qinghai‐Tibet Plateau) under the current and future (2050s, 2070s) three climate scenarios (RCP2.6, RCP4.5, RCP8.5). We found that the climatically suitable habitats for the threatened medicinal plants were primarily found in the eastern, southeast, southern, and some parts of the central regions on the QTP. Moreover, 25% of the threatened medicinal plants would have reduced suitable habitat areas within the next 30–50 years in the different future global warming scenarios. The climatic suitable habitat for 50% of the threatened medicinal plants would migrate to higher altitudes or higher latitudes regions.

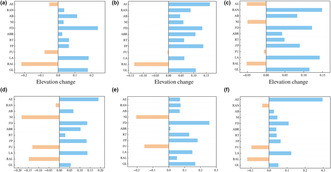

## 1 INTRODUCTION

The interaction and relationship between plants and climate are hot topics in the fields of botany, ecology, and geography (Jiang & Ni, 2005; Wang & Ni, 2006). Over the past 100 years from the Industrial Revolution to the present, human activities have led to drastic changes in the global climate. According to the fifth report of IPCC (the Intergovernmental Panel on Climate Change), human activities have emitted a large amount of greenhouse gases such as CO_2_, leading to an increase in global temperature (IPCC). From 1880 to 2012, the average global surface temperature increased by 0.85°C, and it is projected to increase by 0.3 to 4.8°C by the end of the 21st century (2081–2100) compared to the base period (1986–2005; Hartmann et al., 2013). Under the background of global climate change, climate warming in the coming decades or even centuries may alter the structure and function of terrestrial ecosystems by affecting the distribution of organisms and vegetation composition.

Numerous observations indicate that past climate change has had a significant impact on biodiversity, including changes in species phenology, behavior, distribution and richness, population size and interspecific relationships, ecosystem structure and function, and even leading to the extinction of individual species (IPCC). Biodiversity will be more severely impacted by future global warming. According to the latest assessment by the IPCC report, global warming is predicted to exceed 1.5–2.5°C, increasing the risk of extinction for 20%–30% of the species that have been assessed. In addition, over 2–3°C, 25%–40% of Earth's ecosystem structure and function will experience significant changes (IPCC). Endangered plants are a major component of biodiversity in a region, and rare and endangered plants have a high extinction rate and may face the risk of extinction in the coming period if their species numbers continue to decline (Menges, 1990). The geographical distribution of species is one of the key geographical characteristics of species, and each species has a specific distribution range in the world. The drawing of species distribution maps is an important means of studying species distribution, and plays a crucial part in analyzing the origin and distribution law of species populations (Zhang & Chen, 2003). To further protect global biodiversity and prevent further destruction of ecosystems, it is necessary to systematically understand the relationship between the potential geographical distribution areas of species and climate change, as well as the potential geographical distribution areas of species in future climate change scenarios, and propose adaptive protection strategies.

The Qinghai‐Tibetan Plateau (QTP) has a significant impact on China's and Asia's geographic environment patterns and climatic change. It is recognized as the “driving force” and sensitive area of global climate change, and its response to “greenhouse gases” is more sensitive than other regions (Pan & Li, 1996; Yao et al., 2012, 2020; Zhang et al., 2020). The QTP is one of the geographical regions with the highest number of medicinal plants in China. There are over 2000 kinds of medicinal plants on the QTP, occupying approximately half of the total number of medicinal plants in China (Zhang, 2003). However, with the continuous development of human society and the increase in population, along with the vigorous development of Tibetan medicine, the Tibetan medicine resources in the QTP have been arbitrarily exploited and plundered for a long time, resulting in the destruction of medicinal vegetation habitats, rapid shrinkage of medicinal plant resources, and some species are on the brink of extinction (Law & Salick, 2005; Niu et al., 2021). In addition, climate change with warming as its main feature will have a huge impact on the natural environment, ecosystem, and species distribution in this region, and some endangered wild medicinal plant resources will face greater threats (Allen & Lendemer, 2016; Descombes et al., 2015; Fitzpatrick et al., 2008). Therefore, understanding the potential geographical distribution of threatened species and their suitable habitat status is a prerequisite for effective conservation work (Pearce & Boyce, 2006). However, in reality, the geographical distribution data of many species is scarce, especially for endangered medicinal plants in the QTP.

Many medicinal plants are difficult to survey due to the complexity of the QTP environment, and conservation efforts frequently encounter Wallacean shortfall. Wallacean shortfall refers to our lack of knowledge of species distributions, both because we do not have adequate sampling efforts across multiple regions and lack straightforward methods to generalize from these data to obtain a clear picture of species distributions (Hortal et al., 2015; Lomolino, 2004; Whittaker et al., 2005). The species distribution model (SDM) has been widely used in the Qinghai‐Tibet Plateau to protect endangered medicinal plants as a means of addressing this conundrum (Chen et al., 2022; Xing et al., 2023; Xu et al., 2009). SDMs are numerical models based on Ecological niche theory, also known as ecological niche models (ENMs) or habitat suitability models (HSMs; Guisan & Thuiller, 2005; Searcy & Shaffer, 2016). SDMs use site data and environmental variables to analyze the suitable distribution areas of species and project them into the landscape according to algorithms to reflect the preferences of species for habitats (Guisan & Thuiller, 2005). The running results can be presented in terms of species distribution probability, suitability of distribution areas, and species richness (Guisan & Thuiller, 2005). Currently, species distribution models have become a fundamental research tool in disciplines such as basic ecology and conservation biology. They are also suitable for exploring the patterns of species distribution with climate change in the context of global change, and can effectively predict changes in species distribution (Xu et al., 2009). At present, there are various species distribution models based on ecological niches (Elith et al., 2006), which have been widely used in predicting suitable distribution areas for endangered plants. For example, the maximum entropy model MaxEnt (Abdelaal et al., 2019; Gao et al., 2022; Ma & Zhang, 2012; Zhang et al., 2023; Zhang & Zhao, 2001), the ruleset genetic algorithm model GARP (Li et al., 2023), and the BIOCLIM model (Semwal et al., 2021) and so on. Among them, MaxEnt, the maximum entropy model, is an intensively used prediction model in recent years, and its prediction results are the most accurate. Even though the species distribution data information and environmental variables in the distribution area are incomplete, it can precisely predict the potential distribution area of species (Phillips & Dudík, 2008; Warren & Seifert, 2011). In addition, the stability of the model is good and the predicted results are basically in line with the actual distribution of species (the average AUC value is the largest; Busing & Mailly, 2004).

In this study, the distribution areas of suitable habitats for threatened medicinal plants on the QTP were evaluated on spatial and temporal scales by using the MaxEnt model. The potential impacts of global climate change on endangered medicinal plants on the QTP were evaluated for the first time. The main purpose of this study was: (i) identifying the key areas of suitable habitats for the threatened medicinal plants on the QTP and priority areas for conservation; (ii) estimating the influence of climate change on the suitable habitat of the threatened medicinal plants on the QTP region; (iii) determining the migration direction of suitable habitat for the threatened medicinal plants on the QTP; (iv) comparing changes and trends in future suitable habitat among the different threatened medicinal plants.

## 2 MATERIALS AND METHODS

### 2.1 Occurrence records of the threatened medicinal plant on the QTP

**FIGURE 1:**
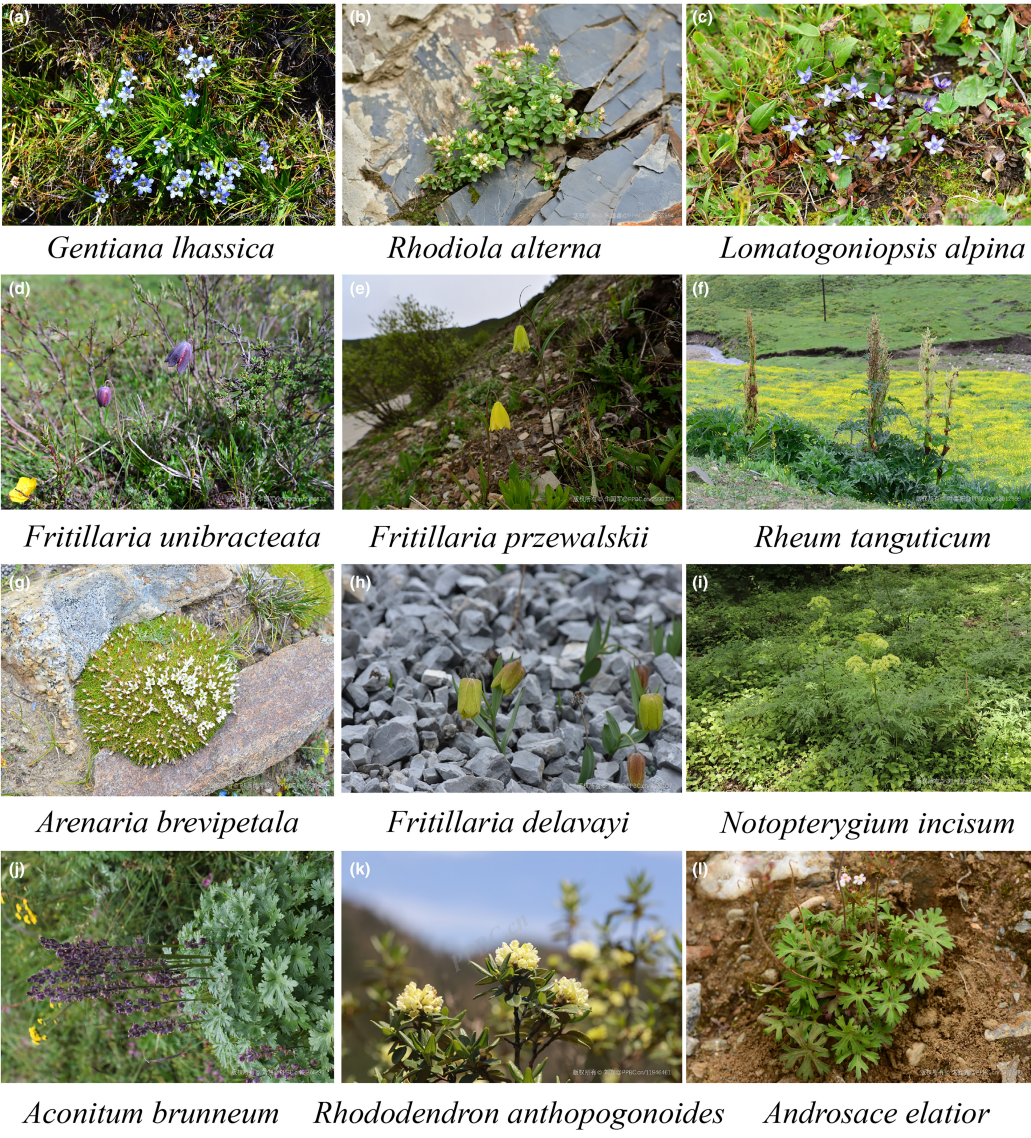
Organism photograph of the 12 threatened medicinal plants on the QTP.

In this study, 12 native threatened medicinal plants on the QTP were used by consulting the books and database such as “Flora Qinghaiica”, “Flora of Tibet”, “List of rare and endangered plants in China”, “Chinese Red List of species”, “List of wild plants under State Key Protection (the first batch)”, The IUCN red list of threatened species (https://www.iucnredlist.org/), China wild plant conservation association, https://www.wpca.org.cn/bhml. Then, these 12 medicinal plants were divided into Critically Endangered (CR), Endangered (EN), Vulnerable (VU), and Near Threatened (NT) levels according to the record of the International Union for Conservation of Nature (IUCN) Red List of Endangered Species (Table 1). Since the NT species is close to or likely to become a threatened category not long in the future, it is included. Organism photographs in the flowering stage of 12 native threatened medicinal plants on the QTP are shown in Figure 1.

**TABLE 1:** The endangered status of the threatened medicinal plants on the QTP.

Abbreviated	Scientific name	Family name	Order name	IUCN level
GL	*Gentiana lhassica*	Gentianaceae	Contortae	CR
RAL	*Rhodiola alterna*	Crassulaceae	Rosales	CR
LA	*Lomatogoniopsis alpina*	Gentianaceae	Contortae	EN
FU	*Fritillaria unibracteata*	Liliaceae	Liliales	EN
FP	*Fritillaria przewalskii*	Liliaceae	Liliales	VU
RT	*Rheum tanguticum*	Polygonaceae	Caryophyllales	VU
ABR	*Arenaria brevipetala*	Caryophyllaceae	Caryophyllales	VU
FD	*Fritillaria delavayi*	Liliaceae	Liliales	VU
NI	*Notopterygium incisum*	Apiaceae	Apiales	NT
AB	*Aconitum brunneum*	Ranunculaceae	Ranunculales	NT
RAN	*Rhododendron anthopogonoides*	Ericaceae	Ericales	NT
AE	*Androsace elatior*	Primulaceae	Primulaceae	NT

The date on the locations of 12 threatened medicinal plants were acquired from field investigation reports from the last 30 years, principally in the specimen libraries, e.g. Chinese Virtual Herbarium (http://www.cvh.ac.cn/), Global Biodiversity Information Facility (https://www.gbif.org/), China National Specimen Information Infrastructure (http://www.naii.org.cn), and the reports in literatures. The total occurrence records for these 12 species are 38, 17, 34, 33, 110, 25, 59, 26,72, 64, 37, and 27, respectively (Table S1). We then used ENMTools.pl (https://github.com/danlwarren/ENMTools) to clip occurrence points to only one observation was reserved in each 30s grid cell (according to the environment variable data below, the resolution of the climate data used for niche modeling, see below), to reduce the sampling bias of the data. Finally, all the remaining occurrence records were applied for the ENM (Table S2).

### 2.2 Climate data collection and environmental variables

The correlation and completeness of variables are the key elements of constructing ENM. In this study, we chose three types of environmental variables, including bioclimatic, soil variables, and topographical (Table 2). Nineteen bioclimatic and altitude variables were downloaded from WorldClim 1.4 (https://world clim.org) for the current period (1960–1990) at a spatial resolution of 30 arc‐seconds (approximately 1 km). Another two topographical variables (slope and aspect) were obtained from the Digital Elevation Model using the 3D analyst tools in the software ArcGIS 10.4. We downloaded the eight soil variables from the National Tibetan Plateau Data Center (https://data.tpdc.ac.cn/zh‐hans/) based on previous studies (Guo et al., 2019; Li et al., 2021; Ru, 2006). Furthermore, the old WorldClim version 1.4 (https://worldclim.org/) was used to obtain projected future climate data. The average values of representative concentration pathways (RCPs) under the future Community Climate System Model were selected to predict suitable area changes in 2050 and 2070. RCPs include RCP4.5 and RCP6.0 (medium greenhouse gas emissions), and RCP8.5 (high greenhouse gas emissions; Thomson et al., 2011). However, the available data on the other two types of environmental variables (soil variables and topographical variables) were lack for the future periods according to the same periods. The 12 species are relatively less affected by topography and soil because they are chiefly located in mountain areas. Therefore, we assumed that these two types of environment variables were constant, as reported in previous studies (Evans et al., 2020; Lv et al., 2021; Zhang et al., 2019).

**TABLE 2:** Environmental indicators used in this paper to model the potential distribution of 12 threatened medicinal plants.

Index	Name	Mean	Unit
Bioclimatic variables	Bio1	Annual Mean Temperature	°C
	Bio2	Mean Diurnal Range	°C
	Bio3	Isothermality (Bio2/Bio7) (×100)	°C
	Bio4	Temperature seasonality (Standard deviation × 100)	CovfV
	Bio5	Max Temperature of Warmest Month	°C
	Bio6	Min Temperature of Coldest Month	°C
	Bio7	Temperature Annual Range	°C
	Bio8	Mean Temperature of Wettest Quarter	°C
	Bio9	Mean Temperature of Driest Quarter	°C
	Bio10	Mean Temperature of Warmest Quarter	°C
	Bio11	Mean Temperature of Coldest Quarter	°C
	Bio12	Annual Precipitation	mm
	Bio13	Precipitation of Wettest Month	mm
	Bio14	Precipitation of Driest Month	mm
	Bio15	Precipitation Seasonality	mm
	Bio16	Precipitation of Wettest Quarter	mm
	Bio17	Precipitation of Driest Quarter	mm
	Bio18	Precipitation of Warmest Quarter	mm
	Bio19	Precipitation of Coldest Quarter	mm
Terrain variables	ASL	Elevation	m
	SLOP	Slope	°
	ASPE	Aspect	°
Top soil variables	PH	Top soil pH (H_2_O)	Log(H+)
	AN	Available nitrogen	mg/kg
	AK	Available potassium	mg/kg
	AP	Available phosphorus	mg/kg
	TN	Total nitrogen	mg/L
	TK	Total potassium	mg/L
	TP	Total phosphorus	mg/L
	Som	Soil organic matter	g/kg

*Note*: There are eight soil and three terrain indicators, and 19 bioclimatic indicators.

To avoid collinearity among various environmental factors resulting in over‐fitting of model prediction results, when using the species distribution model to simulate species distribution, the R language was used to screen environmental variables based on the variance inflation factor (VIF) and Person correlation test, and the accuracy of niche model was improved by reducing the complexity of the model (Ayob et al., 2023; Brauner & Shacham, 1998; Guisan et al., 2002). Relevant research indicates that multicollinearity does not exist when the VIF value between environmental components is less than 10, which is advantageous for model transfer (Zhang & Liu, 2017). In this study, variables with correlations less than 0.8 were first preliminarily screened using packages related to the R programming language (Zhang et al., 2017). Based on the results of this preliminary screening, factors with variance inflation factor VIF values less than 10 were chosen. The final environment factors selected for model construction are list in Table S3.

### 2.3 Optimization of model parameters

The regularization multiplier and feature class are the two most important parameters for establishing the species distribution model using the MaxEnt version 3.4.4 software.

The R package ENMeval was created by Muscarella et al. (2014) and can conduct different user‐defined settings of the MaxEnt model, create data set segmentation using cross‐validation, and improve algorithm accuracy. In this paper, regularization multiplier parameters are set from 0.5 to 4, each interval is 0.5, a total of eight RM parameters. For Feature combination (FC) parameters, the MaxEnt model provides five features: Linear features (L), Quadratic features (Q), Hinge features (H), Threshold features (T), Product features (P). According to these five features, this paper studies five feature combinations: L, LQ, LQH, H, and LQHP (Phillips et al., 2017). To select the combination with lower complexity for modeling, the R package ENMeval was used to adjust RM and FC. The model's complexity under 40 parameter combinations was checked using delta AICc, a 10% training omission rate (avg.OR_10_), and the difference between the training set AUC and the test set AUC (avg.diff.AUC; Zhao et al., 2021).

When delt. AICc is minimum, the model parameters are optimal (Warren & Seifert, 2011; Yang et al., 2022), and the avg.diff.AUC and avg. OR10 are smaller (Shi et al., 2021; Zhao et al., 2021).

It is commonly accepted that the optimal parameter to build the model is the combination of parameters with the lowest increment delt. AICc value. The obtained model of each species had different regularization multiplier and feature class parameters (Table 3; Figure S1).

**TABLE 3:** The selected regularization multiplier (RM) and feature class (FC) parameters for the threatened medicinal plants on the QTP.

Abbreviated	Scientific name	Regularization multiplier	Feature class	Delta. AICc	Avg. diff. AUC	Mean.OR_10_
GL	*Gentiana lhassica*	1	L	0	0.037237	0.250000
RAL	*Rhodiola alterna*	2.5	LQ	0	0.018914	0.166667
LA	*Lomatogoniopsis alpina*	3.5	LQ	0	0.010001	0.150000
FU	*Fritillaria unibracteata*	2	LQH	0	0.063804	0.291667
FP	*Fritillaria przewalskii*	4	LQH	0	0.068375	0.166667
RT	*Rheum tanguticum*	0.5	LQ	0	0.016047	0.062500
ABR	*Arenaria brevipetala*	4	LQ	0	0.050564	0.138393
FD	*Fritillaria delavayi*	3	LQ	0	0.011991	0.150000
NI	*Notopterygium incisum*	0.5	LQ	0	0.017109	0.100000
AB	*Aconitum brunneum*	3	LQH	0		
RAN	*Rhododendron anthopogonoides*	3.5	LQHP	0	0.023414	0.055556
AE	*Androsace elatior*	2	L	0	0.011849	0.229167

### 2.4 Species distribution model

On the base of the relevant parameters ahead, we established the distribution model of the 12 threatened medicinal plants on QTP with MaxEnt version 3.4.4 and projected it to future periods. In the present study, we used 75% of the geographic distribution data as a train and 25% of the geographic distribution data to validate the model. Our analysis set 10 replicates and 5000 bootstrap iterations, with default settings for the other parameters (Hazzi et al., 2018; Morales et al., 2017; Phillips et al., 2006; Radosavljevic & Anderson, 2014). We used the area under the receiving operator curve (AUC) to estimate model performance (Allouche et al., 2006; Swets, 1988). The AUC ranged from 0 to 1. The model with an AUC value greater than 0.9 was regarded as excellent (Guisan & Thuiller, 2005). In addition to AUC, other evaluation indicator, partial ROC was computed (http://shiny.conabio.gob.mx:3838/nichetoolb2/; Osorio‐Olvera et al., 2020; Peterson et al., 2008). It uses an AUC ratio to evaluate the model, and an AUC ratio greater than 1 indicates that the model is better than a random model (Fan et al., 2019; Zhu et al., 2017).

Subsequently, we loaded the arithmetic results from MaxEnt into ArcGIS10.4 to execute suitability classification and visualization and thereby generate the potential distribution of 12 threatened medicinal plants. It was vital to select an applicable threshold when shifting the continuous species suitability prediction results into a Boolean classification of suitable and unsuitable habitats. The maximum test sensitivity plus specificity logistic threshold approach was proved to be superior to other threshold division methods (Poirazidis et al., 2019). Then we used the maximum test sensitivity plus specificity threshold as a threshold or “cutoff” value for each scenario, and habitat suitability was divided into four classes: high suitability, moderate suitability, low suitability, and unsuitability (Table S4).

### 2.5 Distribution barycenter migration of threatened medicinal plants

In the case of further investigating the dynamic migration paths of the 12 threatened medicinal plants, we calculated the centroids of the 12 threatened medicinal plants from their current distribution to their future distribution by using a Python‐based SDM toolbox (Brown, 2014). The analysis centralized the species distribution into an independent central point and established a vector file depicting the magnitude and direction of changes over time (Hu et al., 2015). We observed the distributional shifts by tracking the centroid changes among different SDMs.

## 3 RESULTS

### 3.1 Habitat distribution models for the threatened medicinal plants of the QTP

**FIGURE 2:**
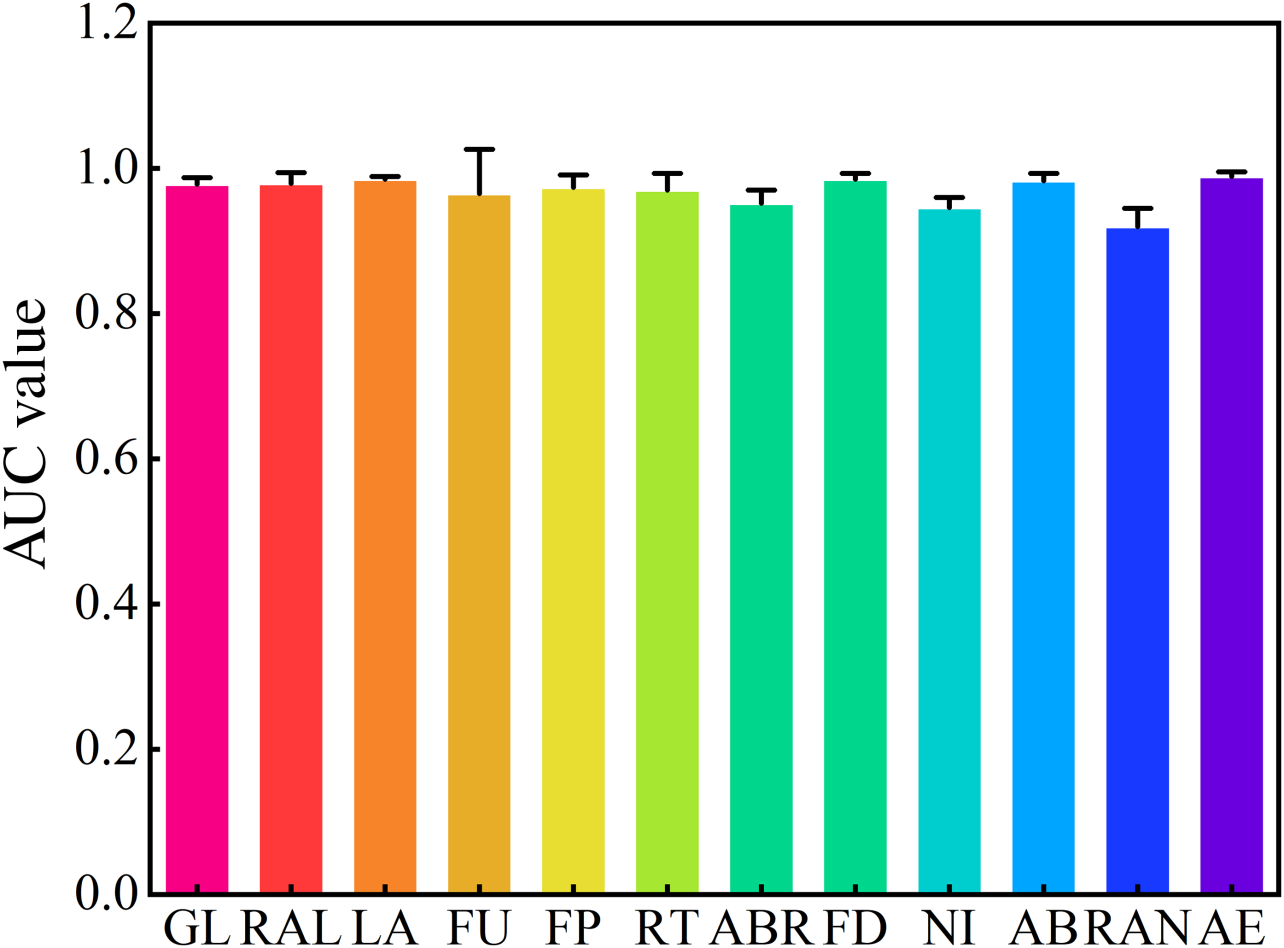
The simulations AUC values of the MaxEnt for the threatened medicinal plants on the QTP.

**FIGURE 3:**
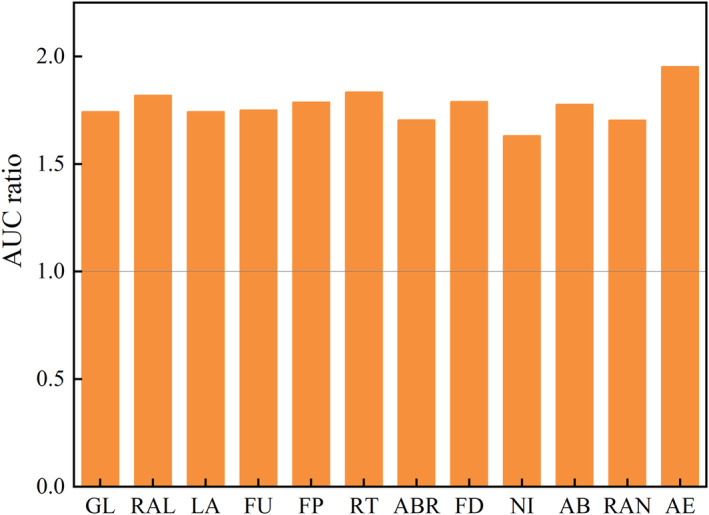
The AUC ratio of the MaxEnt for the threatened medicinal plants on the QTP.

The ROC results showed that the average training AUC value for the 10‐replicate runs in the current and future surpassed 0.9 in all the threatened medicinal plants on the QTP. Furthermore, the standard deviation (SD) values of 10 repetition runs remained below 0.06 in all the threatened medicinal plants on the QTP (Figure 2). In addition, the AUC ratio was greater than 1 for the 12 threatened medicinal plants on the QTP (Figure 3; Table S5). These results indicated that the prediction results of the MaxEnt model were accurate and reliable. Thus, we could conduct a follow‐up analysis.

**FIGURE 4:**
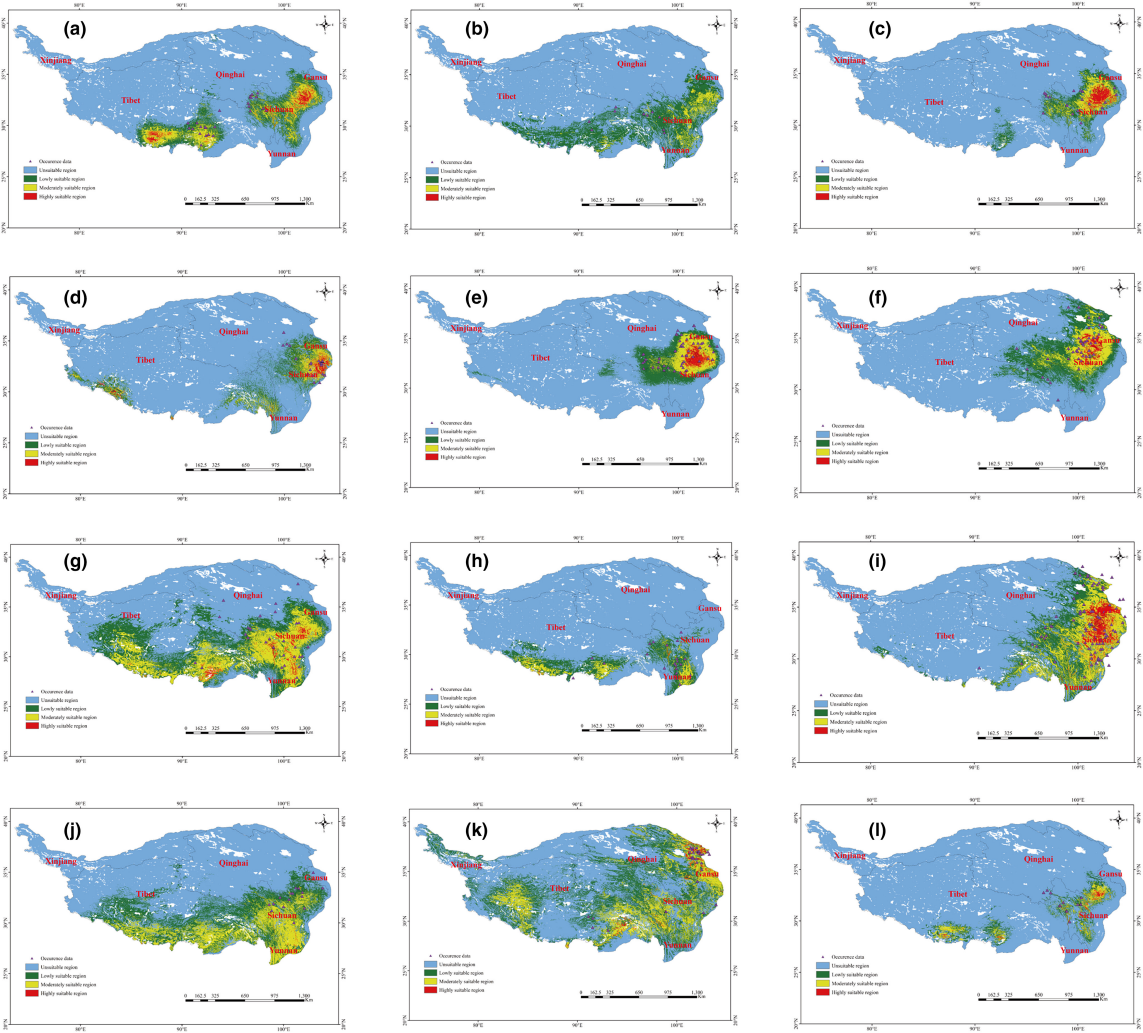
Habitat distribution of the threatened medicinal plants on the QTP. (a) GL; (b) RAL; (c) LA; (d) FU; (e) FP; (f) RT; (g) ABR; (h) FD; (i) NI; (j) AB; (k) RAN; (l) AE.

The models for the present scenario properly predicted the known distribution range of all the threatened medicinal plants on the QTP (Figure 4). As far as CR medicinal plants were concerned, present distribution model predictions results demonstrated that the most suitable habitats for RAL (*Rhodiola alterna*) were different from the GL (*Gentiana lhassica*). GL is located in the southern and southeast regions of QTP, including Shigatse city and Shannan city in Tibet; however, for RAL is only scattered in the southeastern part of the QTP (Figure 4a,b). In addition, the GL had a larger area of high and medium suitable habitat than the RAL (Table S6). The high suitable habitats of the EN medicinal plants, being different from the CR medicinal plants, preferred to inhabit the eastern region on the QTP (Figure 4c,d). These areas include Garzê Tibetan Autonomous Prefecture, Ngawa Tibetan Autonomous Prefecture and Maerkang city in Sichuan Province. The high‐suitable habitats of the VU medicinal plants showed different distribution ranges: FP (*Fritillaria przewalskii*) (Golog Tibtean Autonomous Prefecture in Qinghai Province, Hezuo city in Gansu Province and Maerkang city in Sichuan Province) and RT (*Rheum tanguticum*) were located in the eastern region on the QTP (Golog Tibtean Autonomous Prefecture in Qinghai Province; Figure 4e,f), while ABR (*Arenaria brevipetala*) mainly distributed in the southeast and southern region on the QTP (Shannan and Changdu city in Tibet, Garzê Tibetan Autonomous Prefecture, Liangshan yi autonomous prefecture and Ngawa Tibtean Autonomous Prefecture in Sichuan Province; Figure 4g), and FD (*Fritillaria delavayi*) was found in the southern region on the QTP (Garzê Tibetan Autonomous Prefecture and Liangshan yi autonomous prefecture in Sichuan Province) (Figure 4h). It was worth noting that FP (*Fritillaria przewalskii*) had the largest area, 3.213 × 10^5^ km^2^, of high‐suitable habitat in the threatened medicinal plants (Table S5). Notably, the RAL (*R. alterna*) had the minimum area, only 6.740 × 10^5^ km^2^ and 0.001 × 10^5^ km^2^, of high and medium suitable habitat among the threatened medicinal plants (Table S6).

Among the NT medicinal plants, the largest combine area of high and medium suitable habitat was found in ABR (*Arenaria brevipetala*), with an area of 43.761 × 10^5^ km^2^ (Figure 4k; Table S6). And it was mainly found in the eastern, southeastern, and southern regions of the QTP, containing Yushu Tibet Autonomous Prefecture in Qinghai Province, Shangri‐la city in Yunnan Province, Changdu city, Xigazê Prefecture, and Linzhi city in Tibet and most of the region in Sichuan Province. RAN (*Rhododendron anthopogonoides*) had the second largest combined area of high and medium suitable habitat, with an area of 37.023 × 10^5^ km^2^, which is mainly distributed in the eastern, southwestern and southeastern regions of the QTP, including Haidong city, Haibei city, Huangnan Tibetan Autonomous Prefecture and Golog Tibetan Autonomous Prefecture in Qinghai Province, Gannan Tibetan Autonomous Prefecture in Gansu Province and Xigazê Prefecture, Linzhi city and Changdu city in Tibet, a most region in Sichuan Province. The high and medium suitable habitats of AB (*Aconitum brunneum*) were located in southern and southeastern regions of the QTP, including Gannan Tibetan Autonomous Prefecture in Gansu Province, Ngawa Tibetan Autonomous Prefecture in Sichuan Province and the southern region in Tibet. The combined area of high and medium suitable habitat of AE (*Androsace elatior*) was the smallest, only 6.595 × 10^5^ km^2^, and it was found sporadically in Garzê Tibetan Autonomous Prefecture, Ngawa Tibetan Autonomous Prefecture in Sichuan Province and Xigazê Prefecture and Shannan city in Tibet.

### 3.2 Future suitable habitat fluctuations

**FIGURE 5:**
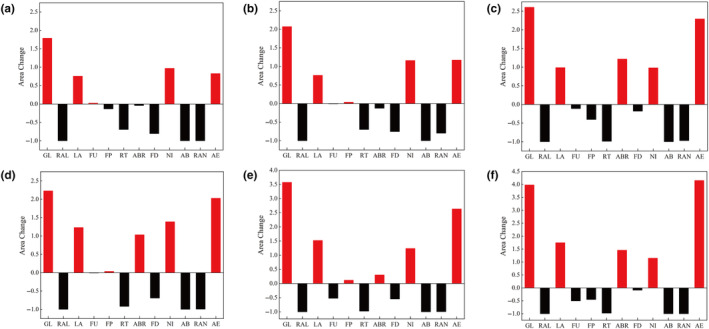
Area changes of high suitable habitat of the threatened medicinal plants in different climate scenarios on the QTP. (a) RCP4.5 climate scenarios in 2050; (b) RCP6.0 climate scenarios in 2050; (c) RCP8.5 climate scenarios in 2050; (d) RCP4.5 climate scenarios in 2070; (e) RCP6.0 climate scenarios in 2070; (f) RCP8.5 climate scenarios in 2070.

The results of future climate model prediction showed that the high and medium suitable habitats of GL, LA, NI, and AE expanded in all future climate scenarios with global warming by comparison with contemporary prediction results (Figure 5; Figures [Link], [Link]; Table S6). Among these medicinal plants, the maximum increasing of high and medium suitability habitats was found in GL, and the fluctuation ranges were 178.71%–397.72% and 23.87%–31.32%, respectively (Figure 5; Figure S2). And they reached the maximum value in the RCP 8.5 and PCR 6.0 climate scenario of 2070, respectively (Figure 5; Figure S2). Notably, the high suitability habitats of RAL were lost in all future climate scenarios, however, the medium suitability habitats increased in all future climate scenarios, and reached a maximum of 332.34% in the RCP8.5 in 2070 (Figure 5; Figure S2). In addition, high suitability habitats for 25% of threatened medicinal plants had a decreased in all future climate scenarios, including FD, RT, and AB (Figure 5). Among these medicinal plants, the high suitable habitat of RT had the largest decrease, and it achieved a maximum of 98.97% in the RCP 8.5 climate scenario of 2050 (Figure 5). The high and medium suitable habitat of the remaining medicinal plants decreased or increased in different future climate scenarios (Figure 5; Figures [Link], [Link]).

### 3.3 Future migratory trends of the threatened medicinal plants

**FIGURE 6:**
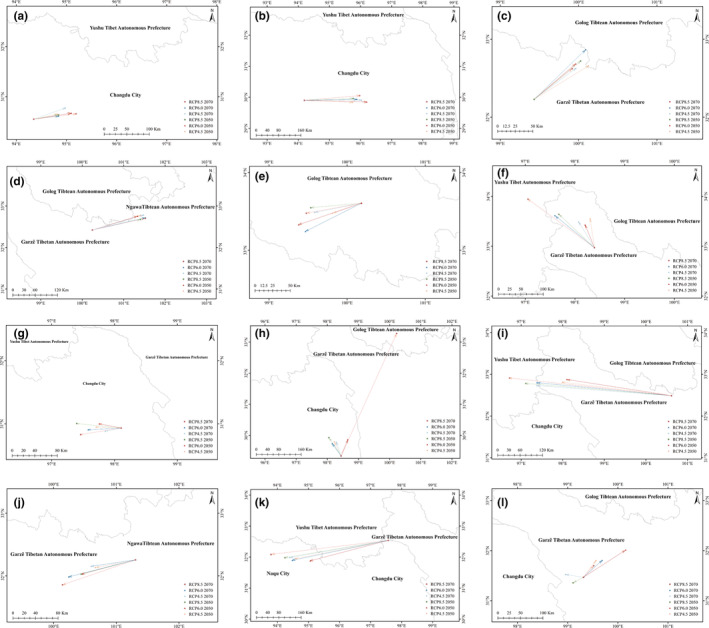
The barycenter migration of the threatened medicinal plants in the future climate scenario.

**FIGURE 7:**
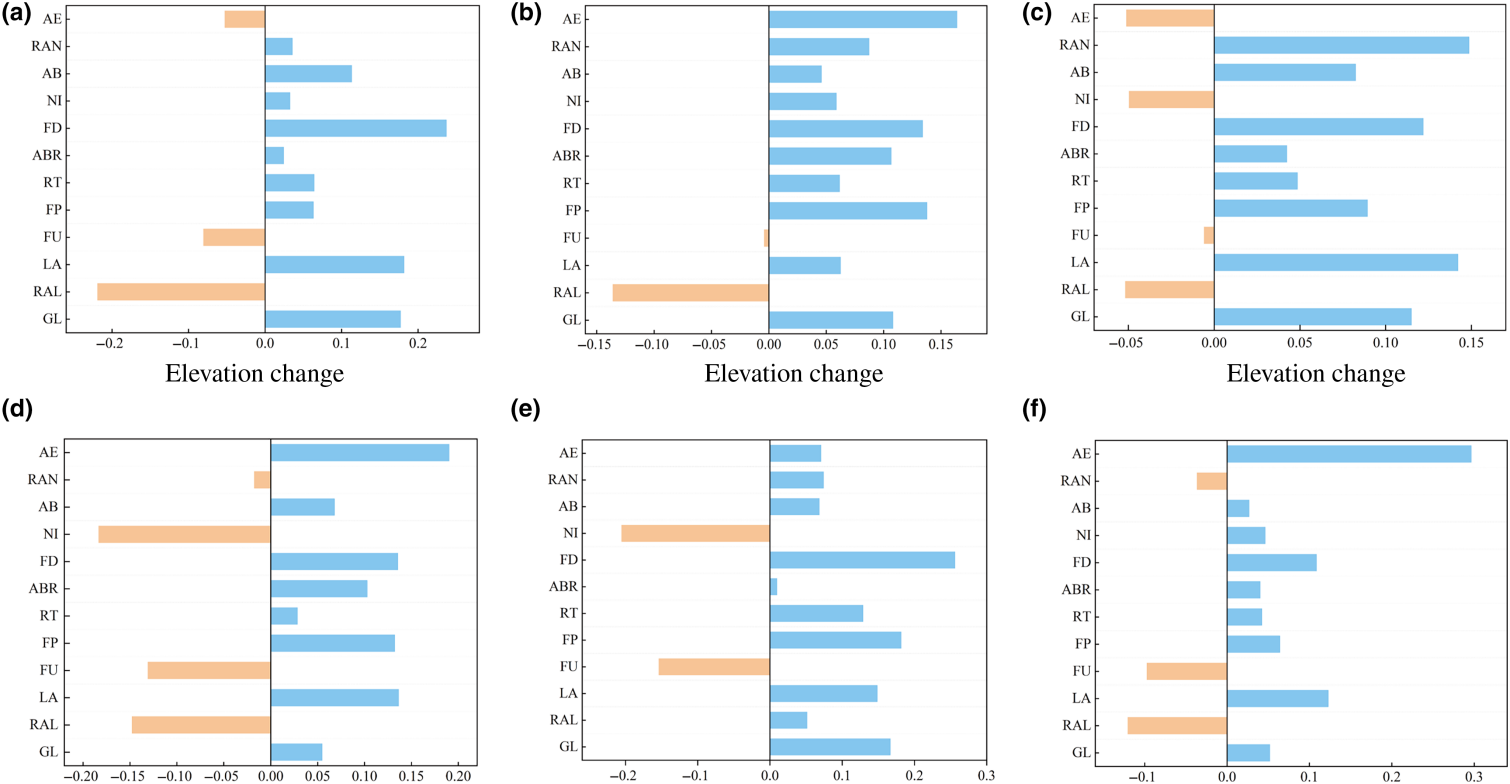
The barycenter elevation change of the threatened medicinal plants on the QTP in the different future climate scenario (a) RCP4.5 climate scenarios in 2050; (b) RCP6.0 climate scenarios in 2050; (c) RCP8.5 climate scenarios in 2050; (d) RCP4.5 climate scenarios in 2070; (e) RCP6.0 climate scenarios in 2070; (f) RCP8.5 climate scenarios in 2070.

Barycenter results of the threatened medicinal plants indicated that the GL, LA, FU, RT, FD, and NI were likely to migrate northward in the future climatic scenario of global climate change (Figure 6a,c,d,f,h,i). On the contrary, the FP, AB, and RAN were inclined to migrate southward in the future distribution model. And, the barycenter of RAL had a trend of eastward migration (Figure 6b), whereas the barycenter of ABR tends to migrate westward in the future climatic scenario of global climate change (Figure 6g). Distance of barycenter migration results indicated that the maximum migration distance of CR medicinal plants were 82 and 193 km, respectively (Table S6). The maximum migration distance of EN medicinal plants was 93 and 127 km, separately (Table S6). As far as VU medicinal plants were concerned, the maximum migration distances of FP, RT, ABR, and FD were 83, 166, 68, and 69 km, respectively. Furthermore, the NI, AB, RAN, and AE in NT medicinal plants had a maximum migration distance of 378, 121, 367, and 101 km, separately (Table S6). In addition, the elevation of the barycenter of LA, FP, RT, ABR, FD, AB, and AE were persistently increased due to future climate change (Figure 7). The FU had a consistently decrease in elevation of barycenter in all future climate scenarios. The elevation of the barycenter of the remaining threatened medicinal plants showed an increase and decrease in different future climate scenarios (Figure 7).

## 4 DISCUSSION

### 4.1 Threatened medicinal plants distribution and habitat fluctuations

Combined the Maxent model with GIS technology, we predicted the potential distribution areas of 12 endangered medicinal plants, and we obtained the spatial distribution pattern and distribution suitability of 12 endangered medicinal plants endemic to the QTP. The AUC values of the models were all more than 0.9, which proved that the prediction results were accurate and could accurately reflect the potential geographical distribution of 12 endangered medicinal plants endemic to the QTP. The results of present suitable habitat distribution models indicated that the low suitability and unsuitability habitats of the threatened medicinal plants were mainly found in the western, northern, northwestern, and most part of the central regions on the QTP, while the habits with high and medium suitability were mainly located at the eastern, southeast, southern and some part of the central regions on the QTP. Previous research indicated that the diversity of medicinal plants on the QTP was mainly concentrated in the eastern and southeastern regions, including northwest Yunnan, western Sichuan, and eastern and southeastern Tibet (Chen et al., 2022; Fan & Bai, 2021; Song et al., 2023; Xing et al., 2023), which was basically consistent with our results. The rich medicinal plant diversity resources in the eastern and southeastern Tibetan Plateau can be attributed to the complex topographic and geomorphic features (Li et al., 2015; Wu et al., 2022), adequate hydrothermal conditions (Favre et al., 2015), and the relatively stable environment during the ice age in this region compared to the inland regions. Therefore, we proposed to establish protected areas in eastern, southeast, and southern regions on the QTP, using the limited protected areas to maximize the protection of more species.

Different species' distribution ranges will be impacted by climate change in different ways. For instance, when Midgley et al. (2003) simulated how climate change might affect species distribution, they discovered that five out of 28 species lost their suitable distribution range and 12 species lost their distribution range under climate change. According to Peterson et al. (2002), 23 of the 28 species migrated and 13 did not overlap geographically as a result of climate change. According to Erasmus et al. (2002), 17% of South African species saw an increase in range, 78% had a decrease in range, 3% showed no change, and 2% went local extinct due to climate change. In Wu's (2011) simulation, the ranges of six species would contract and one species would increase in response to climate change. In this study, under climate change conditions, compared with the present, the potential habitat areas of GL, LA, NI, and AE on the QTP show an overall trend of expansion in the future, while FD, RT, and AB are generally showed a decreasing trend. This could be caused by variations in plant distribution and appropriate climate factors. Certain regions of a plant's acceptable distribution range are no longer suitable due to changes in climate components brought on by climate change, which are beyond the plant's adaptive range. Furthermore, certain plants may no longer be suited for some regions that are acceptable for distribution at the moment due to the varied distribution characteristics of different plants under climate change and the varying adaption variables to harsh climate changes (Woodward, 1987). Furthermore, these plants' dispersal is limited by the existing low temperatures, inappropriate precipitation, and inadequate comprehensive climate indicators. As a result of climate change, these limiting elements may change, allowing these plants to spread further. Naturally, the impact of complicated topography and the regional heterogeneity of climate factors also cause these plants' distribution to vary in distinct ways following climate change.

### 4.2 Trend in migration of barycenter

Climate change plays an important role in species distribution. Temperature and precipitation are the main factors that determine the geographical distribution, growth, and reproduction of species. Rising temperatures, changes in water conditions, and extreme climate events have caused widespread impacts on biodiversity and species distribution patterns (Hu et al., 2022; Lenoir et al., 2008; Ma & Sun, 2018; Wu, 2011). At different time periods in the future, the distribution pattern of most plants and animals in the world shows a clear trend of migration from low latitude and low altitude areas to high‐latitude and high‐altitude areas, accompanied by varying degrees of reduction or expansion (Parmesan & Yohe, 2003; Root et al., 2003). For example, Wilson et al. (2005) found that the average temperature increased by 1.3°C, and the elevation of 16 butterflies in central Brazil increased by 212 m. However, not all species migrated to higher altitudes and latitudes. With the exception of species that are dispersed in the north, Thuiller (2003) discovered that most species migrated northward as predicted in response to climate change. According to Peterson et al. (2002), with climate change, several species in the Capefloristic region migrated west and some to the southwest. According to Shafer et al. (2001), species migrated in various directions as a result of climate change. This study demonstrates that LA, RT, and FD will move to some high latitude and altitude northern regions as a result of climate change. FP and AB will go southward to altitudes that are higher. The ABR will move to higher altitudes in the west. FU will descend to some lower altitudes in the east. This could be the result of regional variations in climate parameters and climate change in the relevant regions, as well as modifications to the patterns of temperature and precipitation distribution brought about by climate change. For example, RT prefers a chilly temperature and great resistance to cold, while FD prefers a cold, cool, humid climate, cold resistance, and to stay away from hot, dry conditions. LA, on the other hand, prefers dampness and shadow, scattered light, and humid habitats. Climate change increases the temperature and changes the precipitation pattern in the current distribution area, which makes them expand to the northern high altitude and some high latitude suitable areas. FU like cold and cool climate conditions, with cold resistance, wet, fear of high temperature, like shade characteristics. Under climate change, the current distribution area will be unsuitable and will expand to the lower altitude area in the east.

In summary, the determination of potential habitats for endangered medicinal plants is of great significance for the collection of wild excellent medicinal provenances and their biological research and provides basic data for an accurate and comprehensive understanding of the distribution and resource reserves of endangered medicinal plants. The high suitability and suitability areas of 12 endangered medicinal plants can be used as key planning and development areas for the cultivation and planting of endangered medicinal plants in the future. However, this article only used climate factors, soil variables, and topographical to simulate the potential geographical distribution areas of the species, and the results obtained may not be suitable for the survival of the species in some areas. In the actual application process, guidance should still be given based on the local hydrological and geological conditions. In future research, more environmental factors such as human interference factors, soil physical and chemical properties, and vegetation type data can be attempted to be added, then conduct on‐site experiments at a small scale to better guide its protection, management, and introduction and cultivation. In addition, this article only selected 12 endangered plants as the research object. It is not yet known whether the potential geographical distribution trend of all endangered plants under future climate change conforms to this law. In the future, more rare and endangered plants need to be added for verification to confirm the universality of the research results.

## 5 CONCLUSION

The conservation of endangered species is based on a systematic understanding of the relationship between the potential geographical range of species and climate change, and the potential geographical range of species under future climate change scenarios. In this study, the suitable habitats for the threatened medicinal plants on the QTP were found in the eastern, southeast, southern, and some parts of the central regions of the QTP, containing the southeastern region in Tibet Province, eastern and central regions in Qinghai Province and northwestern region in Sichuan Province. In addition, 25% of threatened medicinal plants showed a reduction of the area of suitable habitat in different future scenarios. However, the area of suitable habitat of the 33.3% threatened medicinal plants showed an increase to different degrees due to their more responsive ability to respond to environmental stresses. Among these medicinal plants, the most negatively influenced by climate change is RT, which will miss 98.97% of suitable habitat in 2050 when the carbon emissions are high (RCP8.5). And, the least negative affected by climate change is GL, whose area of suitable habitat will increase by 392.72% in 2070 when the carbon emissions are high (RCP8.5). Then, the suitable habitat for 50% of the threatened medicinal plants would migrate to higher altitudes or higher latitudes regions in the climate change future. Based on these findings, in addition to controlling greenhouse gas emissions, we appealed that the endangered species on the QTP with the reduction of the area of suitable habitat should be given more attention in the conservation measures.

## AUTHOR CONTRIBUTIONS

**Lucun Yang:** Investigation (equal); methodology (equal); software (equal); writing – original draft (lead); writing – review and editing (lead). **Xiaofeng Zhu:** Investigation (supporting); software (supporting). **Wenzhu Song:** Investigation (supporting). **Xingping Shi:** Investigation (supporting). **Xiaotao Huang:** Supervision (equal); writing – review and editing (equal).

## CONFLICT OF INTEREST STATEMENT

The authors declare there are no conflicts of interest.

## Supporting information


Figure S1

Figure S2

Figure S3

Figure S4

Figure S5

Figure S6

Figure S7

Figure S8

Table S1

Table S2

Table S3

Table S4

Table S5

Table S6

Data S1

## Data Availability

We used open‐access data from the Global Biodiversity Information Facility database (GBIF, https://www.gbif.org/), Chinese Virtual Herbarium (http://www.cvh.ac.cn/), China National Specimen Information Infrastructure (http://www.naii.org.cn), WorldClim (http://world clim.org), and National Tibetan Plateau Data Center (https://data.tpdc.ac.cn/zh‐hans/).
