# Supplementary figures and images for: Predicting the potential distribution of 12 threatened medicinal plants on the Qinghai‐Tibet Plateau, with a maximum entropy model
Source: Ecol Evol. 2024 Feb 15;14(2):e11042. doi: 10.1002/ece3.11042 (PMC10867876; doi:10.1002/ece3.11042)

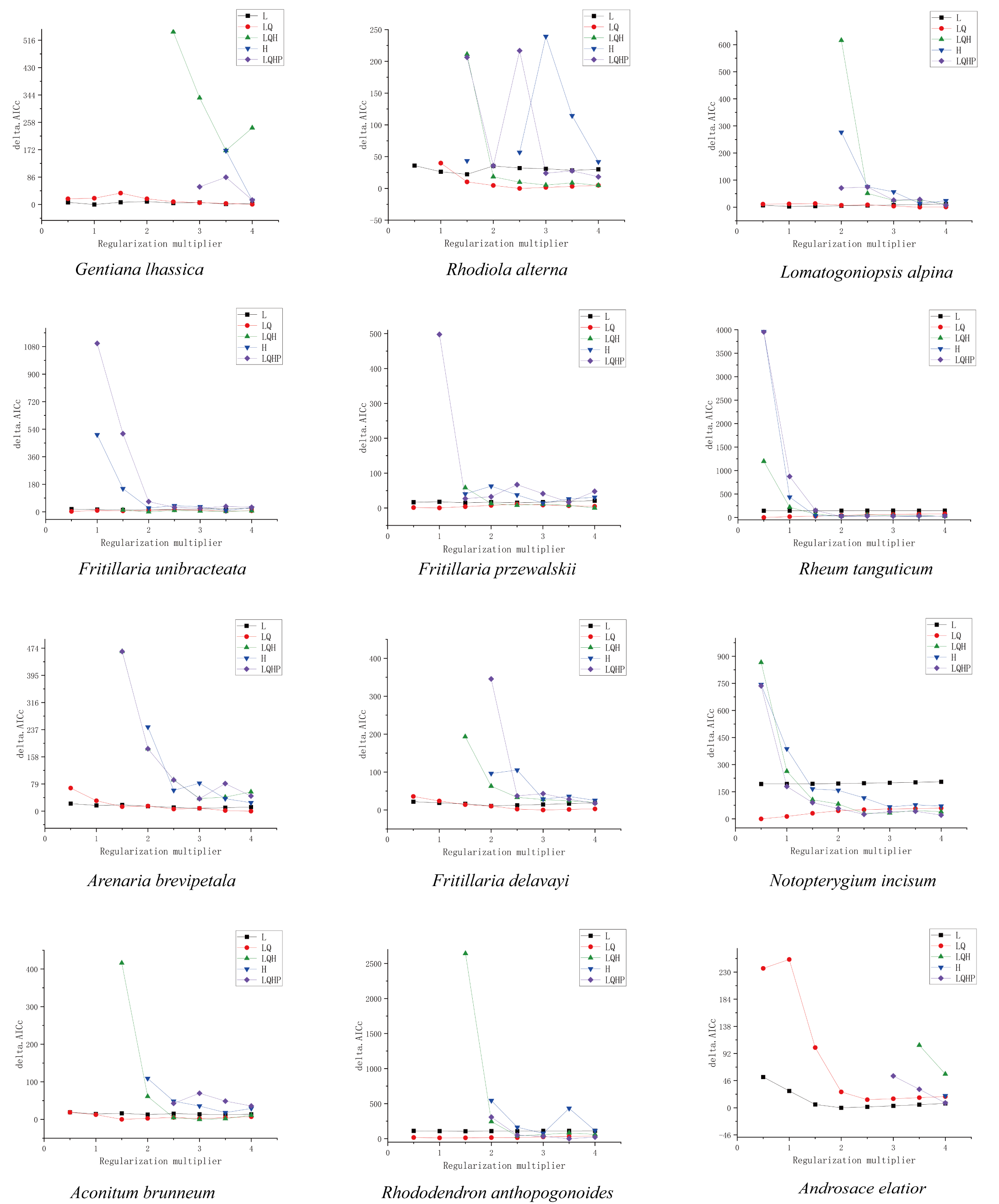

Supplement: Supplementary file 1 — Figure S1 [file ECE3-14-e11042-s010.tif]

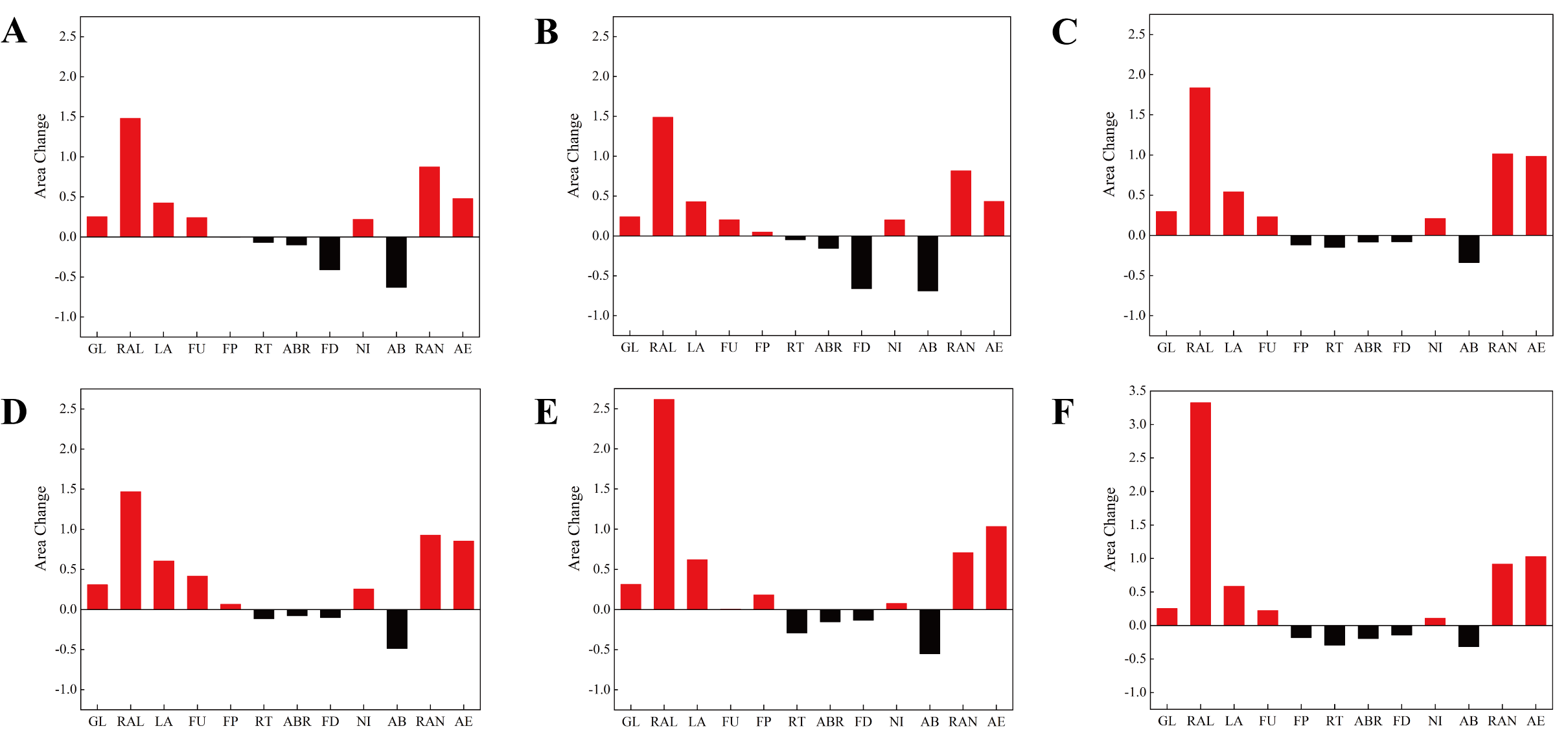

Supplement: Supplementary file 2 — Figure S2 [file ECE3-14-e11042-s012.tif]

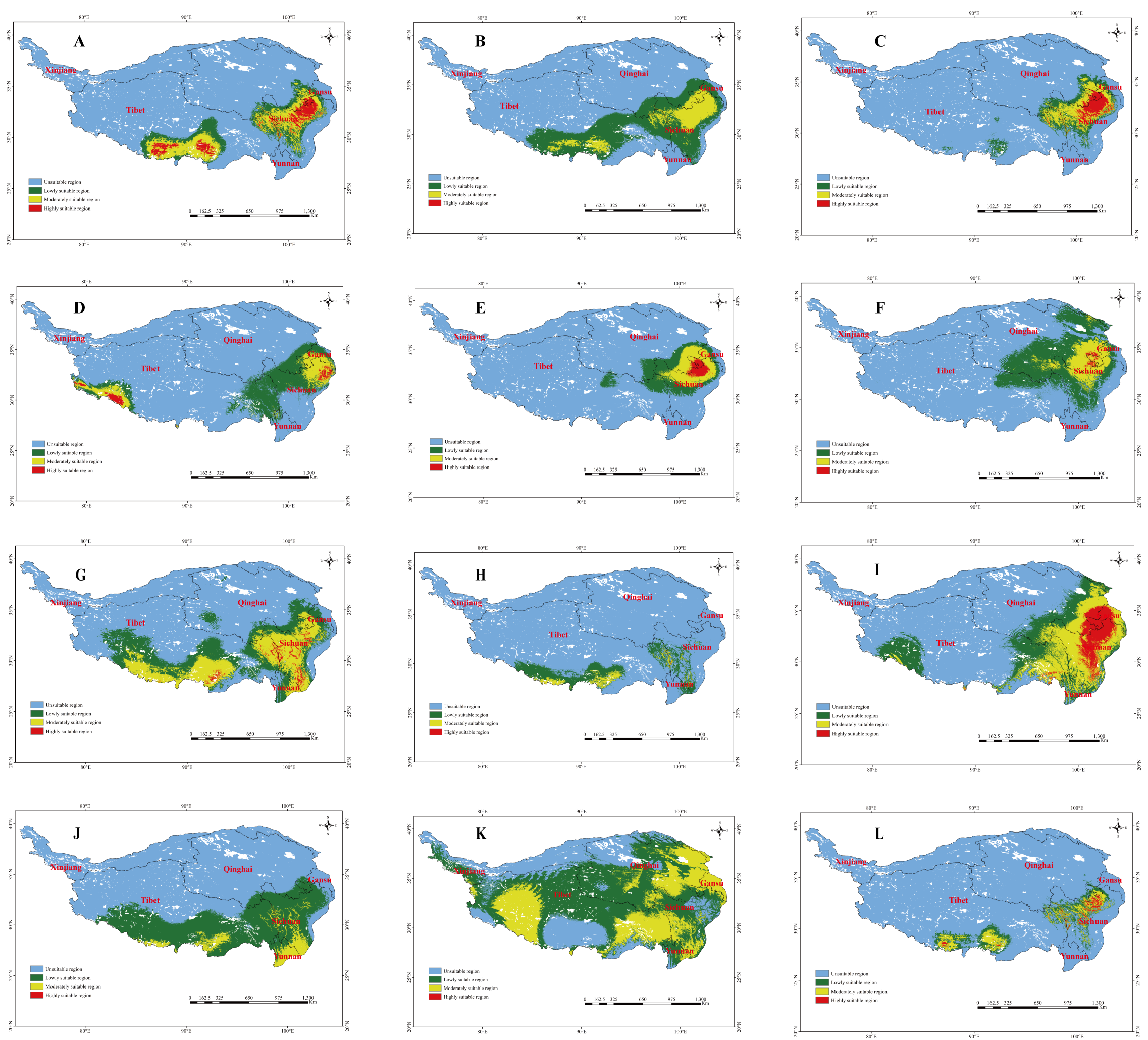

Supplement: Supplementary file 3 — Figure S3 [file ECE3-14-e11042-s002.tif]

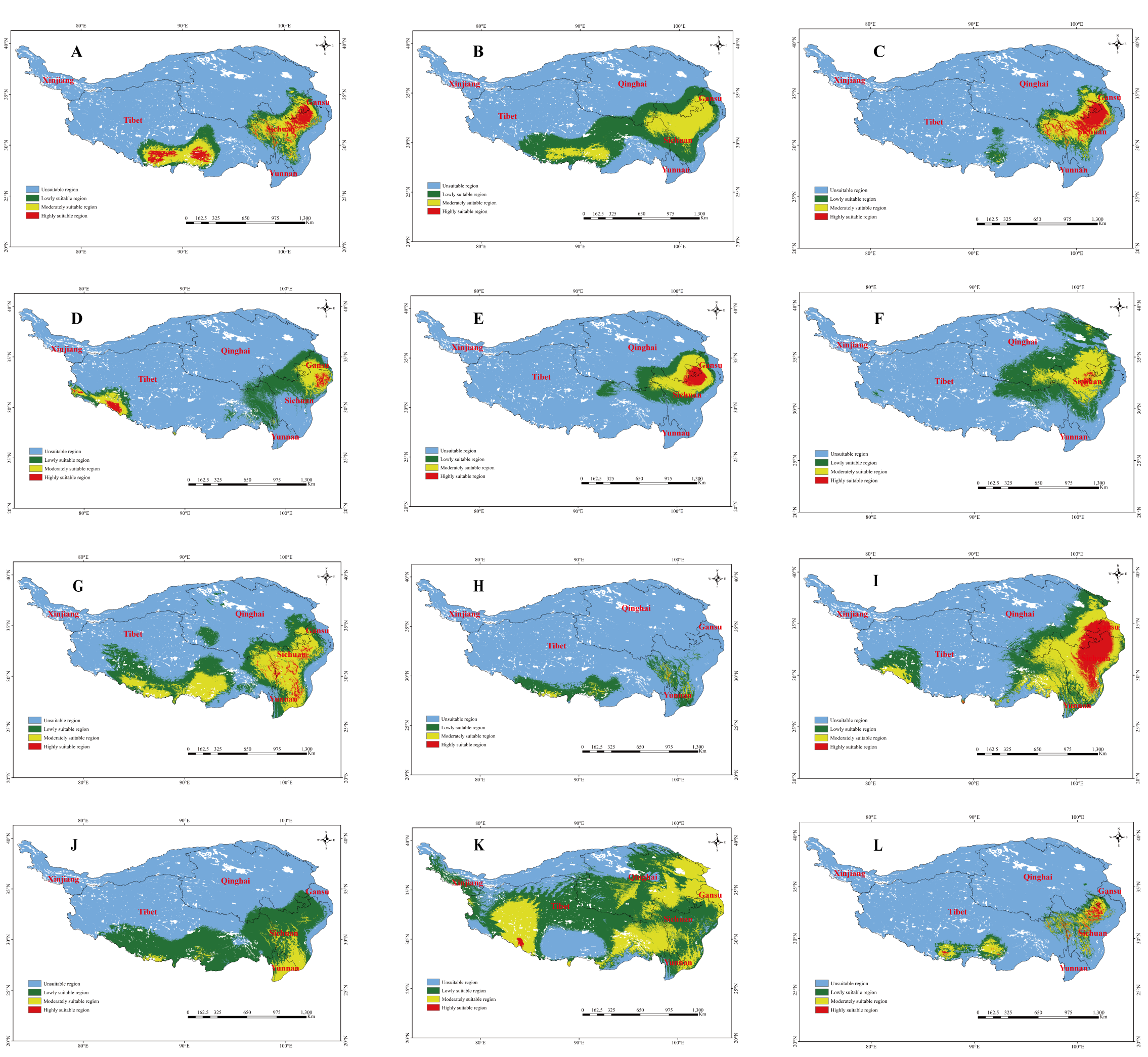

Supplement: Supplementary file 4 — Figure S4 [file ECE3-14-e11042-s015.tif]

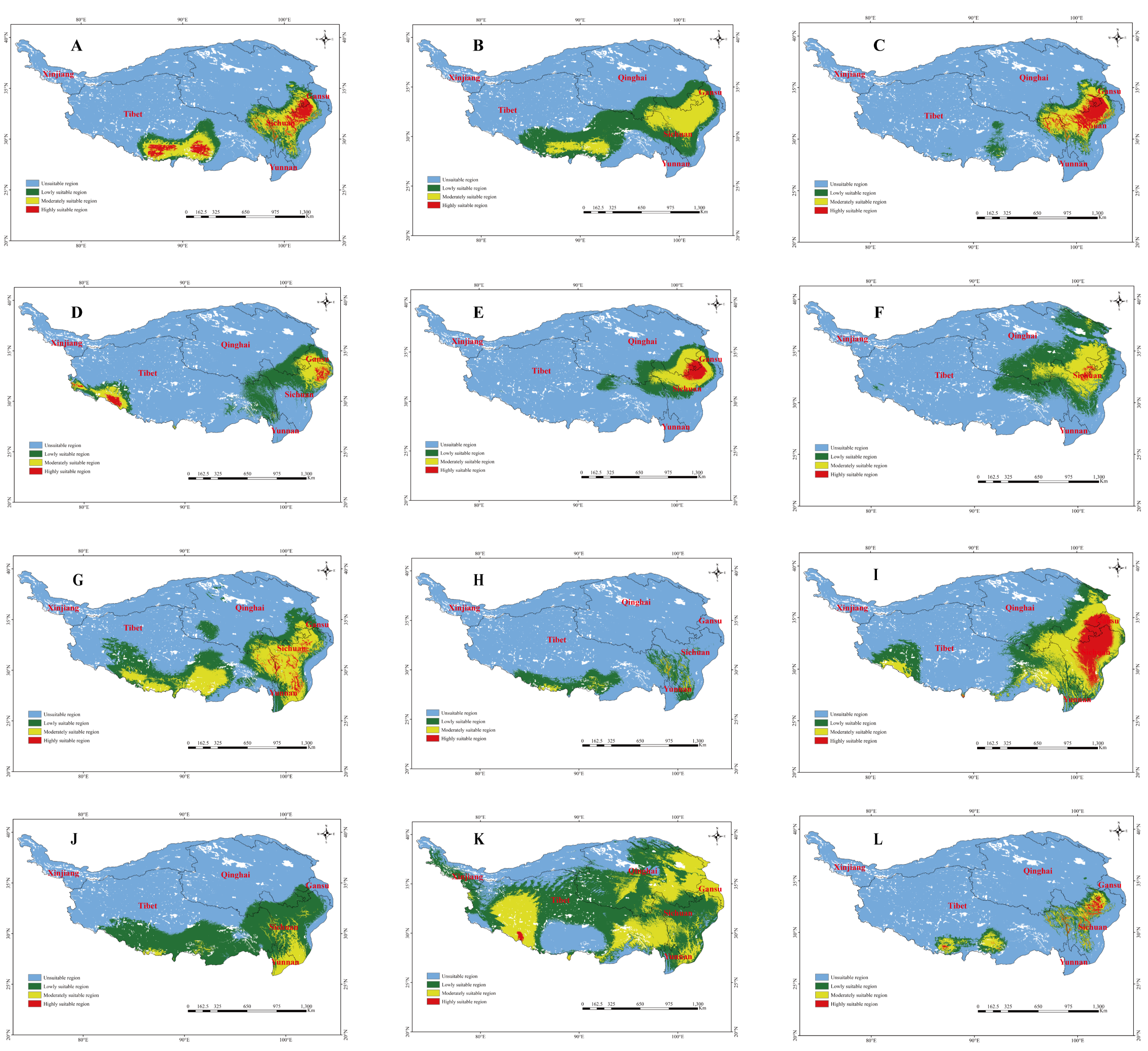

Supplement: Supplementary file 5 — Figure S5 [file ECE3-14-e11042-s008.tif]

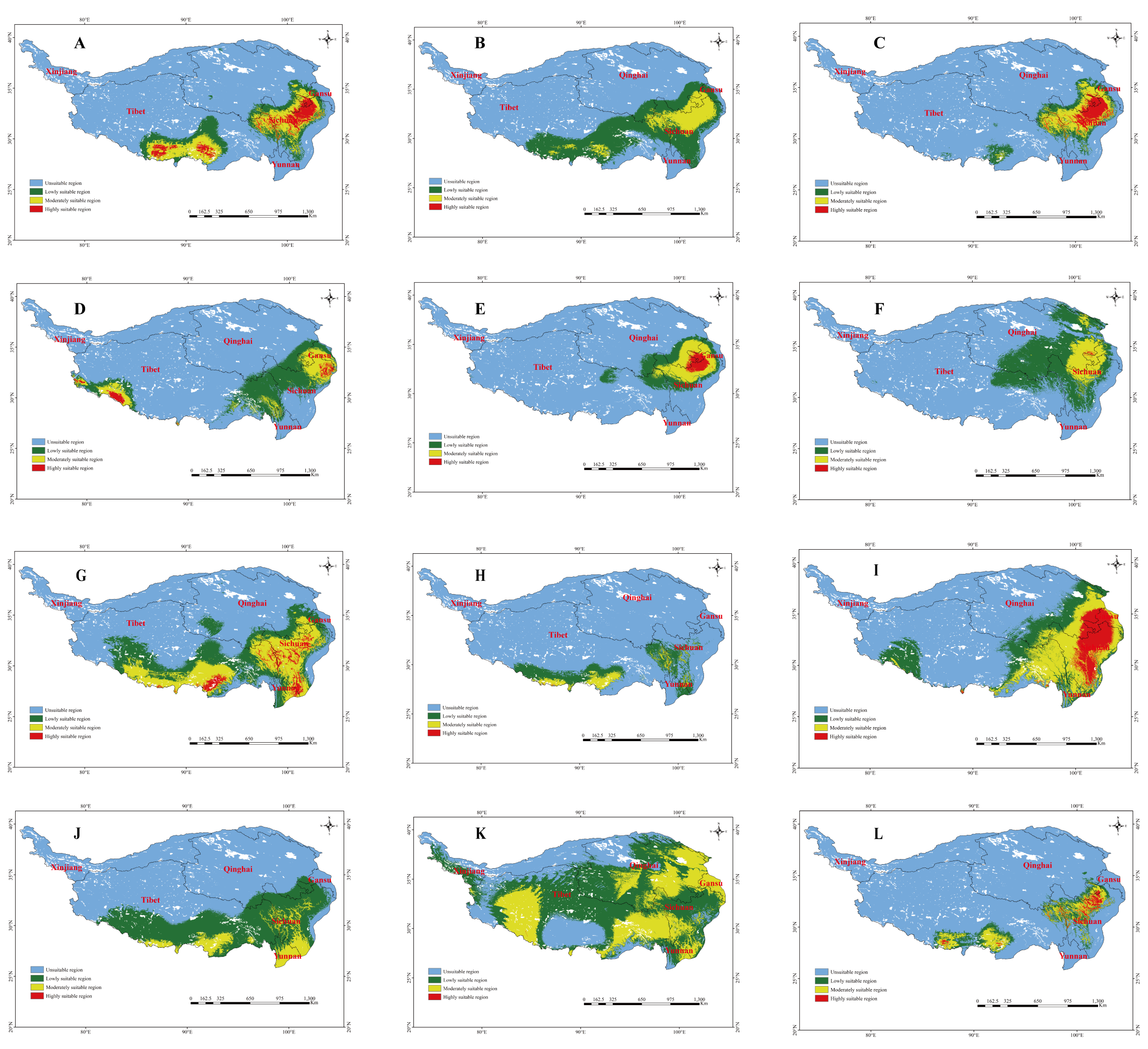

Supplement: Supplementary file 6 — Figure S6 [file ECE3-14-e11042-s006.tif]

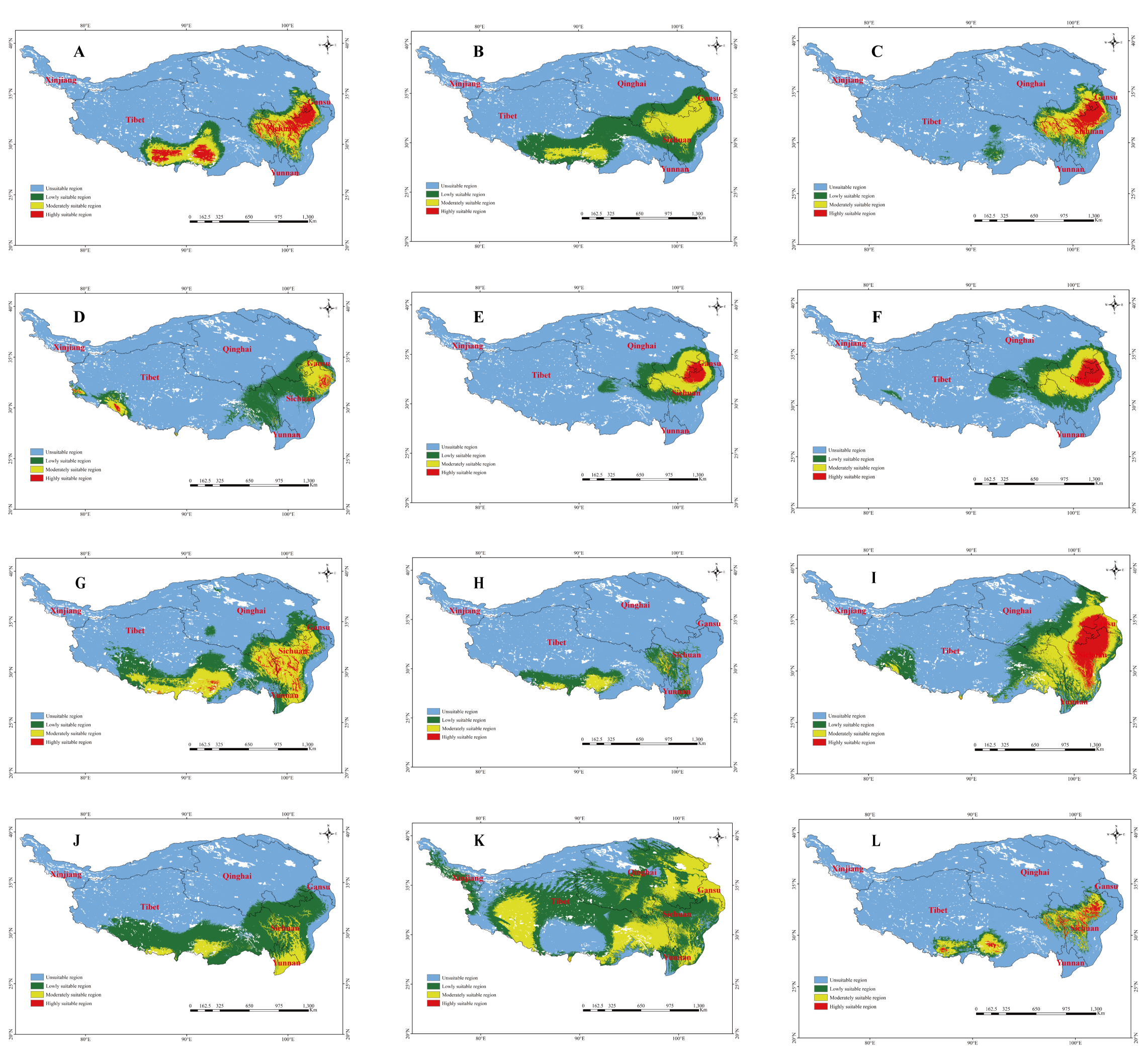

Supplement: Supplementary file 7 — Figure S7 [file ECE3-14-e11042-s014.tif]

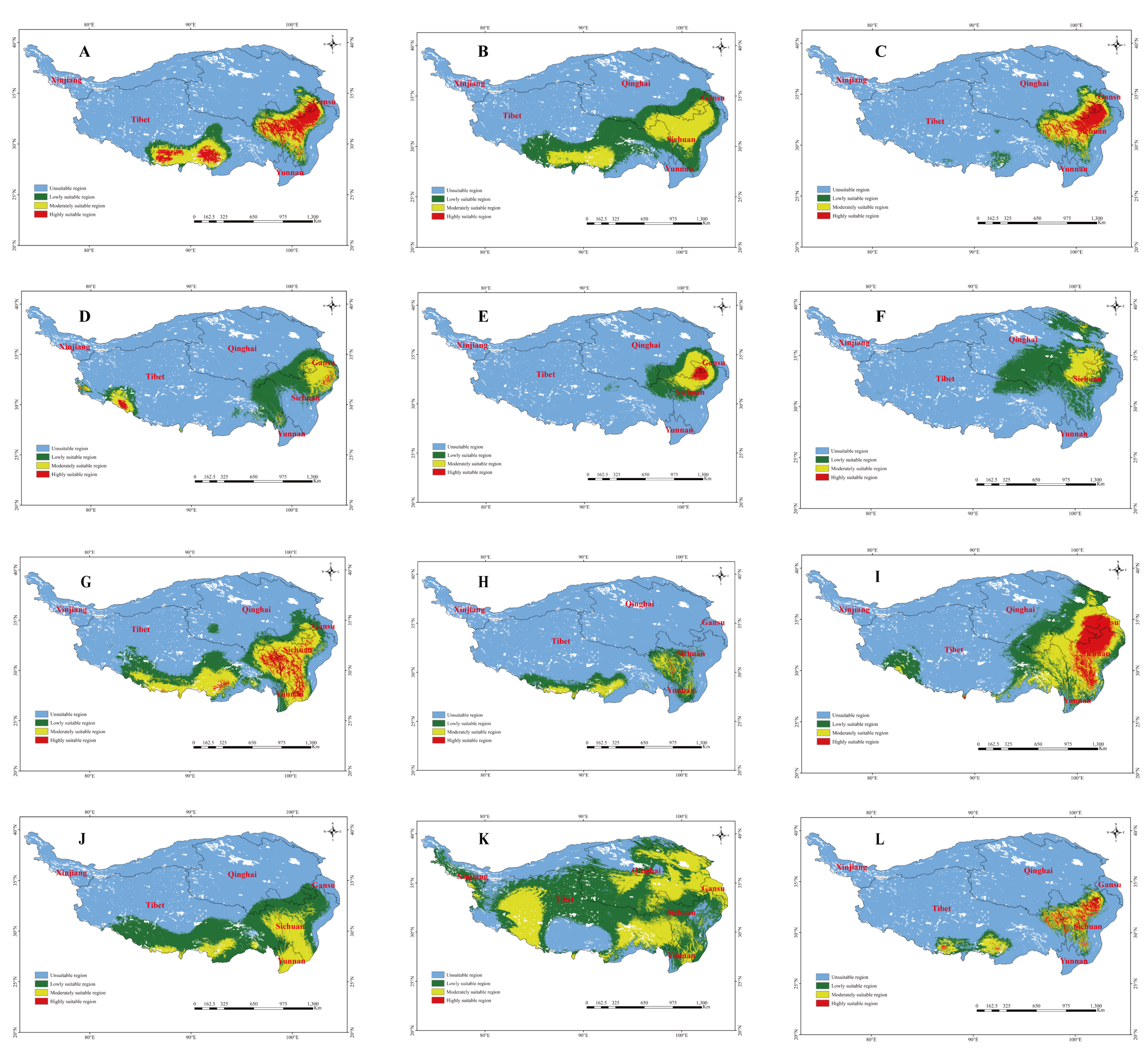

Supplement: Supplementary file 8 — Figure S8 [file ECE3-14-e11042-s003.tif]
