# Supplementary material for: Predicting the potential distribution of 12 threatened medicinal plants on the Qinghai‐Tibet Plateau, with a maximum entropy model
Source: Ecol Evol. 2024 Feb 15;14(2):e11042. doi: 10.1002/ece3.11042 (PMC10867876; doi:10.1002/ece3.11042)
Supplement: Supplementary file 15 — Data S1 [file ECE3-14-e11042-s004.docx]

**Figure S1** DAICc for 12 threatened medicinal plants on QTP.

**Figure S2** Area changes of medium suitable habitat of the threatened medicinal plants in different climate scenarios on the QTP. (A) RCP4.5 climate scenarios in 2050; (B) RCP6.0 climate scenarios in 2050; (C) RCP8.5 climate scenarios in 2050; (D) RCP4.5 climate scenarios in 2070; (E) RCP6.0 climate scenarios in 2070; (F) RCP8.5 climate scenarios in 2070.

**Figure S3** Predicted distribution of the 12 threatened medicinal plants on RCP4.5 climate scenarios in 2050.

**Figure S4** Predicted distribution of the 12 threatened medicinal plants on RCP6.0 climate scenarios in 2050.

**Figure S5** Predicted distribution of the 12 threatened medicinal plants on RCP8.5 climate scenarios in 2050.

**Figure S6** Predicted distribution of the 12 threatened medicinal plants on RCP4.5 climate scenarios in 2070.

**Figure S7** Predicted distribution of the 12 threatened medicinal plants on RCP6.0 climate scenarios in 2070.

**Figure S8** Predicted distribution of the 12 threatened medicinal plants on RCP8.5 climate scenarios in 2070.
